# Burnout and psychiatric morbidity among doctors in the UK: a systematic literature review of prevalence and associated factors

**DOI:** 10.1192/pb.bp.116.054247

**Published:** 2017-08

**Authors:** Udemezue O. Imo

**Affiliations:** 1 Royal Oldham Hospital, Oldham, UK

## Abstract

**Aims and method** To systematically review the prevalence and associated factors of burnout and stress-related psychiatric disorders among UK doctors. An extensive search was conducted of PubMed, EBSCOhost and British medical journals for studies published over a 20-year span measuring the prevalence of psychiatric morbidity (using the General Health Questionnaire) and burnout (using the Maslach Burnout Inventory).

**Results** Prevalence of psychiatric morbidity ranged from 17 to 52%. Burnout scores for emotional exhaustion ranged from 31 to 54.3%, depersonalisation 17.4 to 44.5% and low personal accomplishment 6 to 39.6%. General practitioners and consultants had the highest scores. Factors significantly associated with increase in the prevalence of burnout and psychiatric morbidity include low job satisfaction, overload, increased hours worked and neuroticism.

**Clinical implications** The results indicate a worryingly high rate of burnout and psychiatric morbidity among UK doctors, which could have a huge negative impact on healthcare provision in general. Factors at personal and organisational levels contribute to burnout and psychiatric morbidity, and so efforts made to counter these problems should target both levels.

Doctors have a legal duty broader than that of any other health professional and therefore a responsibility to contribute to the effective running of the organisation in which they work, and to its future direction.^1^ In an environment where their health and well-being is not prioritised doctors sometimes become ill, manifesting features of burnout and/or stress-related psychiatric disorders. Such psychiatric morbidity, or ‘caseness’, is detected using self-reported instruments such as the General Health Questionnaire (GHQ).^2^ Doctors also experience ‘burnout’, which is defined as a syndrome of exhaustion, cynicism and low professional efficacy.^3^ Maslach *et al* described burnout as a prolonged response to chronic emotional and interpersonal stressors on the job, and stated: ‘What started out as important, meaningful, and challenging work becomes unpleasant, unfulfilling, and meaningless. Energy turns into exhaustion, involvement turns into cynicism, and efficacy turns into ineffectiveness’.

Increased prevalence of psychiatric morbidity and burnout has been established in studies from different parts of the world. A study of Italian physicians found an estimated prevalence of psychiatric morbidity to be 25%, and prevalence of burnout on the emotional exhaustion scale of the Maslach Burnout Inventory (MBI) to be 38.7%.^5^ Other studies have reviewed the factors associated with the development and maintenance of psychiatric illness and burnout among doctors. A survey of Australian doctors found that having medico-legal issues, not taking a holiday in the previous year and working long hours were all significantly associated with psychiatric morbidity.^6^ Self-criticism as a medical student was significantly correlated with psychological stress as a doctor in a cohort followed over 10 years by Firth-Cozens.^7^

Burnout among doctors can lead to self-reported suboptimal patient care,^8^ and to major medical errors.^9^ Psychiatric morbidity increases the likelihood of retirement thoughts and retirement preference.^10^ Behavioural responses to burnout established in the literature also include alcohol and drug misuse, physical withdrawal from co-workers, increased absenteeism, arriving for work late and leaving early, and employee turnover.^11^ An extreme reaction to stress can be suicide, even though the pathway to this is complex and multifactorial. A UK survey of suicides between 1979 and 1983 ranked the medical profession as 10th in the list of high-risk professions.^12^

Mental ill health can be found within every workplace in every country. In the UK the total cost to employers of mental health problems among their staff is estimated at nearly £26 billion each year: £8.4 billion from sickness absences and £15.1 billion from reduced productivity at work.^13^ The National Institute for Health and Care Excellence (NICE) found that promoting the mental well-being of employees can yield economic benefits for the business or organisation, in terms of increased commitment and job satisfaction, staff retention, improved productivity and performance, and reduced staff absenteeism.^14^ For the National Health Service (NHS) to reap the benefits described by NICE, priority should be given to employee mental health. However, the constant structural changes to the NHS in England have created instability and lack of job security within the public health workforce.^15^ The Health and Social Care Act of 2012 has placed doctors at the centre of clinical commissioning groups in charge of shaping services and made them responsible for £65 billion of the £95 billion NHS commissioning budget.^16^ This imposes on doctors, especially general practitioners (GPs), a responsibility unlike any before,^17^ one which their training has not prepared them for. The ability to cope with the challenges of working in the NHS and the possibility of stress and burnout were highlighted in the annual meeting of the British Medical Association in 2013,^18^ and are the focus of this review.

Numerous research papers document burnout and stress-related psychiatric disorders in doctors worldwide, but none has presented the results in the form of a systematic review showing the prevalence and associated factors among UK doctors. The overall aim of this review was to redress this by assessing the prevalence of burnout and psychiatric morbidity among UK doctors working in different specialties, and to explore the associated identified factors. The objectives were to review the prevalence of the syndrome of burnout and psychiatric morbidity, to explore the nature of the relationship between burnout and psychiatric morbidity, and to identify other factors associated with the development and/or perpetuation of those conditions.

## Method

### Search strategies

The words ‘burnout’ and ‘doctors’ were put into the search field of the EBSCOhost website specifying the following databases: Academic Search Complete, CINAHL Plus, PsycINFO and PsycARTICLES. Limiters activated were: English language, human, apply related words, and a time limit of January 1993 to December 2013. A total of 562 articles resulted from this, reduced to 489 automatically after duplicates were removed; 28 articles were selected for further analysis, and out of these 9 remained based on the study inclusion and exclusion criteria. Using the same parameters but with the words ‘psychiatric morbidity’ AND ‘doctors’, a total of 97 articles were generated, reduced to 77 after the removal of duplicates, and from these only 1 was selected as new and appropriate. Again using the same parameters but with the words ‘stress’ AND ‘doctors’ NOT ‘nurses’, 3560 articles came up, reduced to 2259 after duplicates were removed; 23 new articles were reviewed in greater detail, and from these 5 new and appropriate articles were selected.

An advanced search on PubMed with the words ‘doctors’ OR ‘physicians’ AND ‘stress’, with a time limit of 1 January 1980 to 15 December 2013 and other limits (human, English language, clinical trial, journal article, reviews, lectures) generated 5973 articles. After careful analysis of the abstracts 28 new articles were identified for more detailed review, and from these 10 were selected as new and appropriate.

Two searches within the group of British medical journals with the phrases ‘burnout and doctors’ and ‘doctors and stress’ with the time limit of January 1993 to December 2013 yielded two new and appropriate papers.

A review of the reference lists of already-identified papers yielded three relevant papers.

**Fig. 1:**
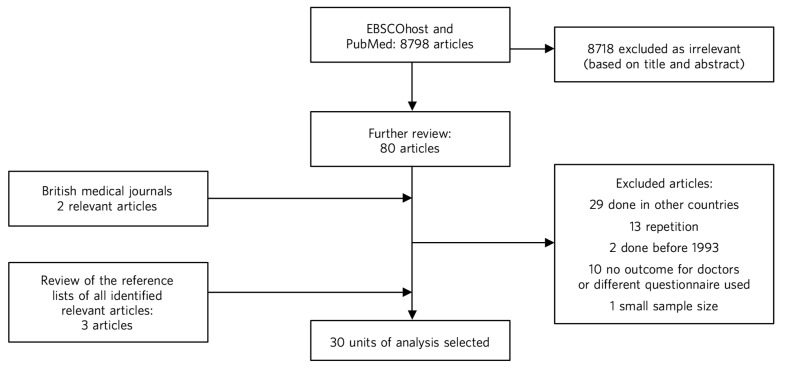
Flowchart of the study selection process.

Altogether, this extensive search yielded 30 relevant papers which were included in the units of analysis for this review (Fig. 1).

### Inclusion criteria

Certain criteria had to be met before a study was included in the units of analysis:

- it had to answer any of the research questions
- for the measurement of the prevalence of psychiatric morbidity the study had to have used any version of the GHQ, and for the prevalence of burnout syndrome only the MBI was considered
- population group – only medical doctors in the UK irrespective of which organisation they work in
- minimum sample size of 50
- published between January 1993 and December 2013
- published in the English language.

### The questionnaires

The GHQ is a well-validated and widely used screening tool for the detection of minor psychiatric disorders (psychiatric morbidity) in the general population.^19^ The GHQ-12 is self-administered and only takes about 5min to complete. It enquires about the experience of psychosocial and somatic symptoms in recent weeks. Each of the 12 items is measured on a 4-point Likert scale. Studies validating the GHQ-12 against standardised psychiatric interviews indicate that a cut-off score of 4 or above indicates a high probability that the individual suffers from a clinically significant level of distress (‘caseness’ or psychiatric morbidity).

The MBI is a 22-item self-report questionnaire, which is well recognised and widely used to measure burnout in relation to occupational stress.^20^ It has three subscales: personal accomplishment (measured by 8 items), depersonalisation (measured by 9 items) and emotional exhaustion (measured by 5 items). Responses are rated for each item according to frequency on a 7-point scale from ‘never’ to ‘every day’. The total score for each subscale is categorised ‘low’, ‘average’ or ‘high’ according to predetermined cut-off scores, based on normative data from a sample of American health professionals. A high degree of burnout is indicated by high scores on the emotional exhaustion and depersonalisation subscales and low scores on the personal accomplishment subscale.

### Data extraction

A simple paper data extraction tool was created in Microsoft Word, and the tables from this have been used to portray the results in the results section. Data were extracted by the author over the months of November and December 2013.

## Results

A total of 30 papers considered relevant and appropriate based on the study inclusion and exclusion criteria were included in this review. Table 1 summarises these papers.

**Table 1:** Units of analysis included in this review

Study	Journal	Running head	Subspecialty/grade
Sharma *et al* (2008)^21^	*Psycho-Oncology*	Stress and burnout in colorectal and vascular surgical consultants	Surgery/consultants

Ramirez *et al* (1996)^22^	*Lancet*	Mental health of hospital consultants: the effects of stress and	Surgery, gastro, oncology, radiology consultants

Wall *et al* (1997)^23^	*British Journal* *of Psychiatry*	Minor psychiatric disorder in NHS trust staff: occupational	Non-specific

Ramirez *et al* (1995)^24^	*British Journal* *of Cancer*	Burnout and psychiatric disorder among cancer clinicians	Oncology/consultants

Sharma *et al* (2007)^25^	*Colorectal Disease*	Stress and burnout among colorectal surgeons and	Surgery/consultants

Kapur *et al* (1999)^26^	*Family Practice*	Sources of job satisfaction and psychological distress in	GP, medical house officer

Guthrie *et al* (1999)^27^	*BJPsych Bulletin*	Sources of stress, psychological distress and burnout	Psychiatry/non-specific

Benbow & Jolley (2002)^28^	*International* *Journal of Geriatric* *Psychiatry*	Burnout and stress amongst old age psychiatrists	Psychiatry/consultants

Orton *et al* (2012)^29^	*BMJ Open*	Depersonalised doctors: a cross-sectional study of 564 doctors	GP

McManus *et al* (2002)^30^	*Lancet*	The causal links between stress and burnout in a longitudinal study of UK	Non-specific

Kirwan & Armstrong (1995)^31^	*British Journal* *of General Practice*	Investigation of burnout in a sample of British general practitioners	GP

Kapur *et al* (1998)^32^	*BMJ*	Psychological morbidity and job satisfaction in hospital consultants	Consultants/junior HO

Coomber *et al* (2002)^33^	*British Journal* *of Anaesthesia*	Stress in UK intensive care unit doctors	Intensive care/consultants

Applet on *et al* (1998)^34^	*British Journal* *of General Practice*	A survey of job satisfaction, sources of stress and psychological	GP

Newbury-Birch & Kamali (2001)^35^	*Postgraduate Medical* *Journal*	Psychological stress, anxiety, depression, job satisfaction	Junior HO

Cartwright *et al* (2002)^36^	*Journal of Clinical* *Pathology*	Workload and stress in consultant medical microbiolo- gists	Microbiology/virology consultants

Caplan (1994)^37^	*BMJ*	Stress, anxiety, and depression in hospital consultants, general	Consultants (non-specific), GP

Burbeck *et al* (2002)^38^	*Emergency Medicine* *Journal*	Occupational stress in consultants in accident and emergency	Emergency medicine/ consultants

Soler *et al* (2008)^39^	*Family Practice*	Burnout in European family doctors: the EGPRN study	GP

Bogg *et al* (2001)^40^	*Medical Education*	Training, job demands and mental health of pre- registration	Pre-registration HO

Upton *et al* (2012)^41^	*Surgery*	The experience of burnout across different surgical specialties	Surgery/consultants

Sochos & Bowers (2012)^42^	*The European Journal* *of Psychiatry*	Burnout, occupational stressors, and social support in psychiatric	Psychiatry, medicine/ senior HO

McManus *et al* (2004)^43^	*BMC Medicine*	Stress, burnout and doctors' attitudes to work are determined	Non-specific

Paice *et al* (2002)^44^	*Medical Education*	Stressful incidents, stress and coping strategies in the pre-registration	Pre-registration HO

Tattersall *et al* (1999)^45^	*Stress Medicine*	Stress and coping in hospital doctors	Non-specific

McManus *et al* (2011)^46^	*BMC Medicine*	Vocation and avocation: leisure activities correlate with professional	Non-specific

Deary *et al* (1996)^47^	*British Journal* *of Psychology*	Models of job-related stress and personal achievement among	Consultants

Thompson *et al* (2009)^48^	*The Clinical Teacher*	Contemporary experience of stress in UK foundation doctors	Foundation doctors

Berman *et al* (2007)^49^	*Clinical Medicine*	Occupational stress in palliative medicine, medical oncology	Oncology and palliative medicine registrars

Taylor *et al* (2005)^50^	*Lancet*	Changes in mental health of UK hospital consultants	Consultants

GP, general practitioner; HO, house officer.

### Findings on prevalence

Seven studies^21,22,24,25,27,30,50^ had quantifiable data on the prevalence of both psychiatric morbidity and burnout (an in-depth analysis of studies reviewed in this paper is included in an online data supplement to this article). Altogether 22 studies reported on prevalence of psychiatric morbidity, and the range was 17–52% (average 31%). GPs and consultants had the highest scores. Fourteen studies had burnout scores, with nine reporting scores as percentages and five as mean scores; one study^28^ had both percentage and mean burnout scores. For emotional exhaustion the scores ranged from 31 to 54.3% and mean scores ranged from 2.90 to 31.26; for depersonalisation the scores ranged from 17.4 to 44.5% (1.95–15.68) and for low personal accomplishment the range was 6–39.6% (4.36–34.21). GPs, consultants and pre-registration house officers had the highest levels of burnout in the studies.

McManus *et al*,^46^ in a UK-wide study carried out in 2009, had the largest sample size at 2845 doctors and reported prevalence of psychiatric morbidity at 19.2%. The other two UK-wide studies with samples of over 1000 cutting across specialties and grades^23,43^ reported psychiatric morbidity prevalence rates of 27.8% and 21.3%, respectively. Taylor *et al*^50^ reviewed 1308 consultants from different specialties and found the prevalence of psychiatric morbidity to be 32%.

One longitudinal study^30^ found no significant increase in the prevalence of psychiatric morbidity over 3 years in a non-specific group of doctors. Another longitudinal study^50^ found a significant increase in psychiatric morbidity and emotional exhaustion among consultants over 8 years.

The only European Union (EU) study looking at the prevalence of burnout in GPs from 12 EU countries^39^ found lower average scores on all burnout scales compared with those of English GPs.

### Findings on associated factors

Job satisfaction was found to be protective against the effect of stress on emotional exhaustion. The number of hours worked, job stress and overload were associated with increased psychiatric morbidity in eight studies. Two studies^22,38^ found that women had significantly higher psychiatric morbidity than men, but three studies^27,34,45^ did not find any association with gender. The personality trait of neuroticism was significantly associated with increase in psychiatric morbidity in three studies,^35,43,47^ while conscientiousness was a protective factor. Psychiatric morbidity was also positively associated with taking work home and with the effect of stress on family life.

Job satisfaction was negatively correlated with burnout in three studies.^21,22,25^ Age was an interesting factor; increased depersonalisation was found in younger doctors in five studies,^21,22,27,29,31^ whereas emotional exhaustion increased with age in two studies.^22,41^ Being single was associated with increased burnout scores, and neuroticism increased burnout significantly in two studies.^43,47^ Increased job stress and workload increased burnout in three studies, with significantly lower emotional exhaustion scores in part-time GPs.

### Findings on the direct relationship between burnout and psychiatric morbidity

Three studies^25,30,46^ found significant positive correlations between psychiatric morbidity as measured by the GHQ, and burnout syndrome. Using the process of casual modelling, McManus *et al*^30^ found that when scores were considered in 1997 and later in 2000, emotional exhaustion increased psychiatric morbidity, and *vice versa*. Personal accomplishment increased emotional exhaustion directly, and increased psychiatric morbidity directly but also indirectly through increasing emotional exhaustion. When other mental health problems were considered, anxiety and depression were found to increase psychiatric morbidity in three studies,^35,37,38^ and depression increased depersonalisation.^41^

## Discussion

The findings indicate that the prevalence of psychiatric morbidity among UK doctors is quite high, ranging from 17 to 52%. This compares unfavourably with the results from a longitudinal survey of people living in private households within the UK, which found an 18-month period prevalence of common mental disorders to be 21%.^51^ Only 4 of the 22 studies that reported on psychiatric morbidity found prevalence of less than 21%,^26,30,32,46^ which is slightly better than 27% found in a study of palliative care physicians in Western Australia.^52^ An earlier study of junior house officers in the UK found psychiatric morbidity in 50% of doctors,^53^ but this was in a period when the working pattern of junior doctors was relatively unregulated. More recent studies of junior doctors contained in this review found the prevalence of psychiatric morbidity to be around 19%.^26,32^ Concern over increasing prevalence of common psychiatric illnesses was borne out by the results from the study which found a 5% increase in morbidity among a cohort of consultants over an 8-year period.^50^

This review also found a high prevalence of burnout among UK doctors measured using the MBI. It lends further support to the growing body of evidence which has found the syndrome of burnout to be prevalent all over the world among health professionals. In a sample of Australian doctors, 24% suffered burnout;^52^ in a New Zealand sample of medical consultants one in five did;^54^ and in a cross-section of Japanese doctors 19% were affected.^55^ This review found even higher rates of burnout, with the prevalence of emotional exhaustion ranging from 31 to 54.3%, which would suggest UK doctors are comparatively more prone to burnout. GPs generally had higher scores for burnout,^29^ particularly in the study of European family doctors,^39^ which found that the only countries in which GPs had higher burnout scores than England were Turkey, Italy, Bulgaria and Greece. Emotional exhaustion among a cohort of consultants was shown to have increased over an 8-year period,^50^ with a prevalence of 41% in 2002.

This review has been able to pool together different studies which report on factors associated with the development and perpetuation of psychiatric morbidity and burnout. Neuroticism was positively and significantly correlated with psychological distress and burnout in three studies.^35,43,47^ Neuroticism refers to a lack of psychological adjustment and instability leading to a tendency to be stress-prone, anxious, depressed and insecure, and it has been shown to negatively predict extrinsic career success.^56^ McManus *et al*,^43^ in a 12-year longitudinal study on a cohort of students who started studying medicine in 1990, found that doctors who are more stressed and emotionally exhausted showed higher levels of neuroticism all through their careers. Neuroticism was also positively associated with perceived high workload. The researchers concluded that neuroticism was not only a correlate but a cause of work-related stress and burnout. Similar findings were noted by Clarke & Singh^57^ in a study looking at the pessimistic explanatory style of processing information, which is a manifestation of neuroticism. In that study neuroticism was shown to positively predict psychological distress in doctors, and the authors recommended that susceptible doctors should be offered cognitive-behavioural therapy (CBT) to alter their explanatory style.

In an editorial titled ‘Why are doctors so unhappy?’ Richard Smith stated that the most obvious cause of doctors' unhappiness was that they feel overworked and under-supported.^58^ Job stress, feeling overloaded and the number of hours worked were positively linked to psychiatric ‘caseness’ and burnout in many of the studies in the present review, and this cut across specialties and grades. A General Medical Council (GMQ survey^59^ of doctors in training found that 22% felt their working pattern leaves them short of sleep at work, and 59% said they regularly worked beyond their rostered hours. Increasing job stress without a commensurate increase in job satisfaction was associated with the presence of psychiatric morbidity, and job satisfaction was also positively correlated with illness in six of the reviewed studies ^21,22,25,34–36^ Another significant finding was the correlation between psychiatric disorders and burnout, with the two feeding off each other, leading to worsening outcomes.

The public health importance of these findings cannot be overemphasised. GPs are at the frontline of healthcare delivery in the UK, and around 90% of all NHS contacts take place in primary care, with nearly 300 million GP consultations a year.^60^ The estimated total number of GP consultations in England rose from 217.3 million in 1995 to 300.4 million in 2008, with a trebling of telephone consultations, and with the highest consultation rates among the growing population of elderly individuals.^61^ Increased live births of over 110 000 over the past 10 years,^62^ and an ageing population^63^ have contributed to the pressure felt by services in general. However, in spite of the increased demand on primary care services, the proportion of the NHS budget that is spent on general practice has slumped to record levels, and GPs report that this has compromised the quality of care they can provide.^64^ Under these circumstances, the added expectation from the UK Department of Health that GP surgeries should open for longer hours and should expand patient choice will undoubtedly lead to even more psychological distress and burnout among GPs.

A government-driven emphasis in the NHS on performance management and targets increases job demands and stress among managers,^65^ and increases psychiatric morbidity among doctors. The current climate of austerity in the UK, and the expectation that doctors should continue to provide high-quality care to patients within an NHS intending to make £20 billion worth of savings,^66^ further expose doctors to burnout and stress. Psychiatrists are already having to deal with the expected increase in demand for mental health services stemming from the economic downturn,^67^ and the increase in suicide rates^68^ among the working-age population. Psychiatrists are particularly vulnerable to burnout, and patient suicide is a factor significantly associated with stress and burnout in this group ^69^

Burnout among doctors can affect the entire public health workforce because as a syndrome it is considered ‘contagious’.^4^ With the push for doctors to take up leadership positions at every level within the NHS a burnt-out doctor can negatively affect the entire healthcare delivery system. Unhealthy coping strategies in response to burnout and stress were identified in this review: these include retiring early, taking work home, taking it out on family, mixing less with friends, and avoidance, all of which work against the development of a healthy work-life balance.

### Limitations

Some key limitations are worth highlighting. First, all the studies were cross-sectional surveys using questionnaires sent to the participants online or by post. Response rates varied, with some as low as 17%, and only in half of the studies was effort made to increase the response rate by sending reminders or repeat questionnaires. Non-response bias could have affected the results. Second, although the MBI was used in all the studies examining burnout, different versions of the MBI were utilised. With the GHQ some studies used the 28-item version but most used the 12-item version. The cut-off for ‘caseness’ using the GHQ also differed between studies and ranged between ⩾3 and ⩾5. However, these differences may not have significantly affected the overall findings given that a study to validate the two versions of the GHQ found no difference between them, and also established that the different cut-off for ‘caseness’ did not affect the questionnaire's validity.^2^

The cross-sectional method used for the surveys makes it difficult to draw a firm conclusion on the outcomes from a cause and effect perspective. Also, the number of potential confounders for the presence of burnout and common psychiatric disorders is vast and cannot be controlled for in surveys alone.

The fact that this literature review ends in 2013 may be considered a limitation, but the hope is that this paper will trigger more research in this area, and the author's intention is to update the literature review by 2023.

### Recommendations

Doctors are ultimately responsible for the quality of care they provide at any time, and they need to be aware of their own vulnerability to burnout and psychiatric illness, and of their impact on patient care. Traditionally, doctors take pride in working a lot of hours,^70^ and are 3 to 4 times less likely to take days off sick compared with other health professionals;^71^ this combination is a recipe for burnout. A whole list of support networks is available on the GMC website,^72^ and doctors should be encouraged to utilise these. However, there is a ‘culture of fear’ among doctors regarding the GMC, and 96 doctors, a lot of whom had mental health problems, have died by suicide since 2004 while being investigated by the GMC.^73^ A lot more work is therefore needed to make the most vulnerable doctors feel supported.

At an organisational level, approaches designed to reduce the workload of doctors should be prioritised. Changes to doctors' contract of service should reflect an understanding of the impact of work-related factors on the health and well-being of doctors, and any such contract should contain the necessary protections to reduce the experience of psychiatric illness and burnout. The benefits of a healthy workforce on the quality of care provided in the NHS cannot be overstated.
